# Shockwave Lithotripsy for De-Novo Urolithiasis after Kidney Transplantation: A Systematic Review of the Literature

**DOI:** 10.3390/jcm12134389

**Published:** 2023-06-29

**Authors:** Clara Cerrato, Victoria Jahrreiss, Carlotta Nedbal, Francesco Ripa, Vincenzo De Marco, Manoj Monga, Amelia Pietropaolo, Bhaskar Somani

**Affiliations:** 1 Department of Urology, University Hospital Southampton NHS Trust, Southampton SO16 6YD, UK; clara.cerrato01@gmail.com (C.C.); victoria.jahrreiss@meduniwien.ac.at (V.J.); carlottanedbal@gmail.com (C.N.); f.ripa92@gmail.com (F.R.); ameliapietr@gmail.com (A.P.); 2 Department of Urology, Comprehensive Cancer Center, Medical University of Vienna, Vienna General Hospital, 1090 Vienna, Austria; 3 Urology Unit, School of Urology, Faculty of Medicine, Università Politecnica delle Marche, 60121 Ancona, Italy; 4 Department of Urology, Fondazione IRCCS Ca’ Granda Ospedale Maggiore Policlinico Milano, 20122 Milan, Italy; 5 Department of Urology, Azienda Ospedaliera Universitaria Integrata di Verona, 37126 Verona, Italy; vzodemarco@gmail.com; 6 Department of Urology, University of California San Diego, San Diego, CA 92037, USA; mamonga@health.ucsd.edu; 7 European Association of Urology-Young Academic Urologists (EAU-YAU), Urolithiasis and Endourology Working Group, NL-6803 AA Arnhem, The Netherlands

**Keywords:** allograft, renal transplant, de-novo urolithiasis, SWL, shockwave

## Abstract

Background: Allograft urolithiasis is an uncommon, challenging, and potentially dangerous clinical problem. Treatment of allograft stones includes external shockwave lithotripsy (SWL), flexible ureteroscopy and lasertripsy (fURSL), or percutaneous nephrolithotomy (PCNL). A gap in the literature and guidelines exists regarding the treatment of patients in this setting. The aim of this systematic review was to collect preoperative and treatment characteristics and evaluate the outcomes of post-transplant SWL for stone disease. Methods: A systematic search in the literature was performed, including articles up to March 2023. Only original English articles were selected. Results: Eight articles (81 patients) were included in the review. Patients were mainly male, with a mean age of 41.9 years (±7.07). The mean stone size was 13.18 mm (±2.28 mm). Stones were predominantly located in the kidney (*n* = 18, 62%). The overall stone-free rate and complication rates were 81% (range: 50–100%) and 17.2% (14/81), respectively, with only one major complication reported. A pre-operative drainage was placed in eleven (13.5%) patients. Five patients (6.71%) required a second treatment for residual fragments. Conclusions: SWL is a safe and effective option to treat de novo stones after transplantation. Larger studies are needed to better address allograft urolithiasis management.

## 1. Introduction

Renal transplantation is a preferred and effective treatment for advanced renal failure [1,2]. Over 2 million patients receive dialysis or renal transplants worldwide, and this represents only 10% of patients who need treatment [2,3]. Allograft urolithiasis is an uncommon clinical problem, affecting between 0.2 and 1.7% of the grafted population [4,5,6,7]. Despite their rare incidence, allograft stones can lead to significant morbidity, an increased risk of infection, and deterioration of renal function, as transplanted patients have a solitary functioning kidney [8,9]. Overall, stones can form de novo or be transplanted within the donor kidney, which is called “donor-gifted renal stones” [8,9]. Due to the possible complications, donor lithiasis was until recently considered a contraindication to transplant [2]. A computed tomography (CT) scan of the donor prior to transplantation has increased rates of diagnosis, and now stones are usually treated prior to transplant to increase the donor pool [10].

Urolithiasis in transplanted patients is challenging. In fact, due to denervation, patients might be asymptomatic or present with mild abdominal discomfort due to the stretching of the overlying fascia and abdominal musculature caused by hydrodistension, or they might just present with acute deterioration of renal function [11,12]. To date, a gap in Guidelines exists according to the best treatment option for donor or allograft lithiasis [13]. Therapeutic strategies include external shockwave lithotripsy (SWL), flexible ureteroscopy and lasertripsy (fURSL), or percutaneous nephrolithotomy (PCNL), according to the stone’s characteristics, anatomical conditions, surgeon preferences, and patient counseling. However, endourological management is generally challenging and needs a high-volume center. The principal challenges to URS relate to the anatomy of the kidney and ureter and a higher risk of ureteral perforation [14]. With PCNL, the pelvic location might cause adjacent organ injury, the presence of perirenal reactive tissue might cause difficulties in tract dilation and decrease the nephroscope’s mobility, and finally, immunosuppression might impair wound healing and be associated with postoperative sepsis [15]. SWL has the advantage of being a less invasive procedure but nevertheless has some limitations due to the possible masking of pelvic bones [16].

The aim of this systematic review was to collect preoperative and treatment characteristics and evaluate the outcomes of post-transplant SWL for stone disease.

## 2. Methods

### 2.1. Evidence Acquisition: Criteria for Considering Studies

Inclusion criteria:

- English-language articles;
- Studies including at least five patients of any age;
- All articles report on allografts treated with SWL for de-novo urolithiasis.

Exclusion criteria:

- Non-English articles;
- Studies examining treatment for non-urolithiasis conditions;
- Studies reporting on donor (ex-vivo) urolithiasis;
- Studies reporting on transplant urolithiasis treated with other minimally invasive procedures (URS or PCNL);
- Case reports, review articles, historical cohort studies, laboratory studies, and animal studies.

### 2.2. Search Strategy and Study Selection

The systematic review was performed following the Preferred Reporting Items for Systematic Reviews and Meta-analysis (PRISMA) statement [17]. PubMed, Scopus, and Web of Science were searched systematically for English-language articles published up to March 2023 on SWL for the treatment of urolithiasis in allografts. The search terms used included: “ESWL”, “shockwave”, “SWL”, “renal transplant”, “allograft”, “urolithiasis”, “kidney calculi”, and “kidney stone disease”. Boolean operators (AND, OR) that were used to refine the search. This review has been registered in PROSPERO (registration number CRD42023437850).

### 2.3. Study Selection and Data Extraction

Two investigators (C.C. and V.J.) independently screened all titles and abstracts from the literature overview to identify the eligible studies and then evaluated the full-text manuscripts to determine the final selected articles. Any discrepancies were resolved by consultation with the senior investigator (B.S.). The following variables were extracted from each study: journal and year of publication, study type, number of included patients, patients’ demographics (male-to-female ratio, mean age), stone size and location, drain insertion rate, stone-free rate (SFR), complications (rate, type, and grade according to the Clavien Dindo classification [18], management), and stone recurrence. Data were collected using Microsoft Excel (Microsoft Corporation, Washington, DC, USA), version 16.71. The quality of evidence was assessed, and bias was analyzed using the grading of recommendations, assessment, development, and evaluation assessment tool [17]. MOOSE criteria were assessed for the inclusion of observational studies (Supplementary Table S1).

## 3. Results

### 3.1. Study Selection and Characteristics

**Figure 1:**
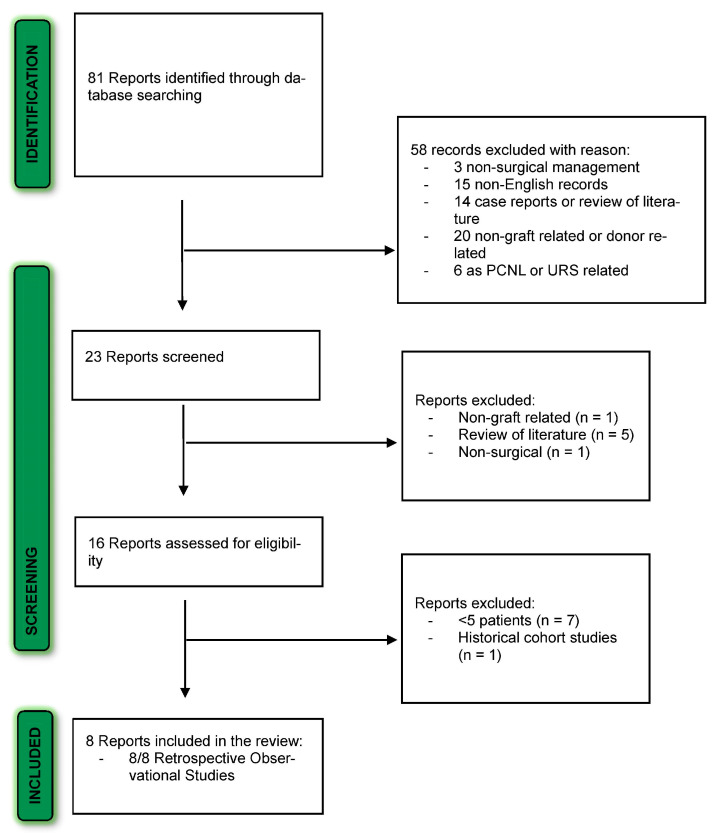
PRISMA flow diagram for the systematic review.

The PRISMA diagram shows the literature search results (Figure 1). After excluding reports that were out of the scope of our systematic review or not in English, we identified 23 overall articles for screening. Of them, 15 full-text articles were reviewed and assessed for eligibility. Eight articles met the inclusion criteria and were included in the final review.

### 3.2. SWL for De-Novo Urolithiasis in Transplanted Patients

There were a total of 81 patients, with a mean age of 41.9 years (±7.07 years). Table 1 and Table 2 show the summary and the outcomes of the stones of the included studies. Overall, for the studies that reported it, there were more males, with a male-to-female ratio (i.e., male:female) of 18:11 [9,12,13]. Preoperative imaging was either ultrasound (USS) [5,6,19,20,21], CT scan [5,6,22], USS and CT scan [23], or kidney, ureter, and bladder plane X-ray with USS [22]. In most cases, the diagnosis was incidental, with the most commonly cited symptom at presentation being macrohematuria, followed by urinary tract infection (UTI) and deterioration of renal function. More rarely, patients are presented with hydronephrosis, anuria, and/or pain. Stones were most commonly located in the kidney (*n* = 18, 62.1%), followed by the ureter (*n* = 10, 37.9%). The mean stone size was 13.18 mm (±2.28 mm). Only three studies reported the type of ultrasound generator used [19,20,24]. Eleven patients (13.5%) had a stent or nephrostomy placed before the procedure for hydronephrosis. Post-operatively, one study reported the routine use of a ureteral stent for 1–3 post-operative days [19], and another study reported the routine use of alpha-lytic therapy [23]. The overall SFR was 81% (range: 50–100%) during the respective follow-up time frame. Five patients (6.71%) required a second procedure, either URS (*n* = 1) [23] or PCNL (*n* = 4) [21,22]. Two studies reported recurrences in a total of three patients (3.7%) [6,20]. The overall complication rate was 17.2% (*n* = 14), and complications reported were graded as follows: seven Clavien I (transient hematuria, conservative management), six Clavien II (Urinary tract infection requiring antibiotic therapy), and one Clavien IIIa (steinstrasse requiring a nephrostomy placement and a subsequent reintervention).

### 3.3. Quality Assessment of Studies

Overall quality of evidence was graded as “very low” and the risk of bias was “very serious”, as detailed in Table 3. All eight studies were retrospective in their design, with their inherent bias as shown.

## 4. Discussion

Renal transplants are commonly performed for an increasing number of patients to treat end-stage renal disease. De-novo urolithiasis in this population usually develops between 9 and 102 months after transplantation [21,25,26], with a median presentation time of 30.5 months [6]. Urolithiasis has been considered a contraindication to transplant and a cause of significant morbidity in the post-transplant setting [27]. Treatment options include expectant management, SWL, fURS, and PCNL. SWL might be used for small caliceal stones with minimal risk of complications. However, previous reports showed lower stone-free rates [16]. In this systematic review, we wanted to assess the challenges and outcomes of SWL in the setting of renal transplant patients.

In most cases, diagnosis was made by USS, found incidentally during follow-up [5,6,19,20,21,22,23] or with mild abdominal discomfort due to kidney denervation [11,12,23,28]. According to some authors, a CT scan might be useful, especially if a treatment is planned [23]. A plain X-ray is not recommended since often the stone is situated over the pelvic bones [21,23].

As for urolithiasis in the general population, imaging plays a critical role in the diagnosis, follow-up, and urological management. Even if USS is particularly helpful as an initial diagnostic tool, a CT scan offers higher sensitivity and specificity (>95% and >96%, respectively) [29,30,31,32,33]. Additionally, in the setting of an allograft, a CT scan might offer important anatomical information with respect to the surrounding organs and to the allograft itself. Some authors indeed recommend performing a CT scan before the procedure, independent of the imaging used at the diagnosis [23].

Many of the clinical features of urinary stones after transplantation differ from those of non-transplant patients. Due to ureteral and renal denervation, the typical renal colic or pain is usually absent, and patients are usually asymptomatic or mildly symptomatic. Additionally, in the transplanted population, there is an overall tendency for more stones in the renal location than the ureteral location [22,24]. The frequent follow-up imaging to which these patients are subjected may explain why these stones are more frequently detected by chance in the kidney before their migration into the ureter.

When symptomatic, patients usually present with gross hematuria, acute renal failure in the case of obstructive stones, or a UTI in the case of infection [27]. Mild symptoms might be caused by the stretching of the nociceptors present in the overlying fascia, too. Indeed, due to the pelvic location, the hydrodistension induced by obstruction causes fascial stretching and subsequent abdominal discomfort. Alternatively, hydrodistension may present as a painless mass at the transplant site. Rarely, the presentation resembles acute rejection or acute tubular necrosis [25]. According to these findings and to the EAU Guidelines [13], the most common symptoms were macrohematuria, followed by UTI, and increased creatinine with or without anuria [5,6,19,20,21,22].

**Figure 2:**
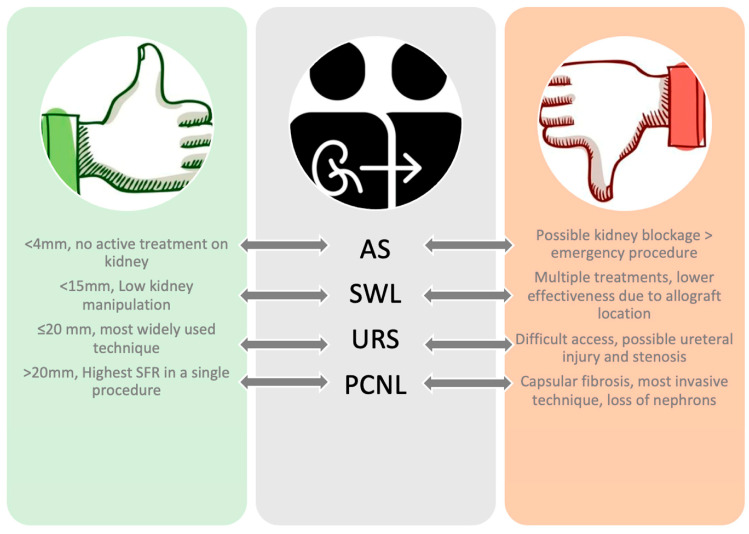
Pros and cons of endourological techniques in allograft patients.

Once diagnosed, there are several treatment options, each with pros and cons (Figure 2). Obstructive stones with fever, uremia, decreased urinary output, and refractory pain should be promptly treated, as prompt removal of the stone causes no significant changes in renal allograft function [25,34]. Non-obstructive stones can be treated conservatively, as previous studies reported spontaneous passage with no change in renal function for stones <4 mm [6,23,35,36]. Conservative management could be divided into medical treatment and expectant management. In the case of expectant management, follow-up must be not only clinical but also laboratory and radiological due to the absence of innervation of the transplant kidney [24,37,38]. Medical treatment for larger stones seems feasible, even if few reports are available in the literature. Romero-Vergas et al. have reported the complete resolution of a staghorn stone after adequate drainage of the pelvicalyceal system with a ureteral stent and applying medical treatment, coupled to a single SWL session [39]. When urate stones are identified, some patients might benefit from urinary alkalinization both for prevention and treatment of stones [6,40]. In a series of 19 patients with uric acid nephrolithiasis after renal transplantation, it was reported that patients were successfully treated with medical therapy, including daily water intake above 3000 mL to maintain the urine volume in the range of 2000–3000 mL/d, urine alkalinization with sodium bicarbonate, oral allopurinol, analgesics, and antispasmodics [16]. When an intervention is planned, any of the contemporary management options should be offered to transplanted patients; as per guidelines, no treatment is superior to the other [13]. Indeed, all techniques present their pros and cons, which must be discussed with the patients.

For fURS, the new anatomy of the ureteroneocystostomy is not always compliant for the insertion of the ureteroscope. The ureter is usually implanted at the dome of the bladder, high in the posterior or anterior wall, with an angle between the ureteral orifice and the scope that is often <120° [23]. For this reason, ureteral access might be difficult, and once accessed, the lack of soft tissue architecture presents an additional risk of perforation [23,27,41,42]. However, fURS might have an advantage in the treatment of stones in difficult anatomical locations, like the middle or lower calyx [40]. In a prospective, uncontrolled study, Timsit et al. compared the ureterocystostomy surgical technique to pyeloureterostomy, which is usually considered a salvage procedure [43]. According to their results, pyeloureterostomy may represent a valuable alternative to ureteroscystostomy, allowing further endourological access to the allograft urinary system, avoiding vescicoureteral reflux, and thus minimizing the risk for UTI [43]. Thus, in the event of a short native ureter or as per the surgeon’s choice, this urinary anastomosis might be considered.

Relatively to PCNL, on the one hand, patients might have difficulties healing due to immunosuppressants [19,44], and on the other, for surgeons, the procedure might be challenging due to the formation of an inflammatory capsule around the transplant, limiting the pyelocaliceal dilation, with additional fibrosis limiting the range of nephroscope movements [12,21]. However, PCNL is the technique that potentially offers the highest SFR in a single procedure for larger stones [40,45].

SWL offers lower kidney manipulation and might be used for renal stones <1.5 cm and for ureteral stones too, as reported by previous large cohort studies [5], with a SFR of 78.8% [46]. In our analysis, SFR was assessed at 80% (range: 50–100%) and the complication rate at 17.2%. Most complications were minor and treated either conservatively [6,24] or with antibiotics [6,23]. Only a single major complication (steinstrasse) requiring nephrostomy tube placement was reported [6].

Even if SWL shows good SFR and complications, it is not devoid of disadvantages. Due to the anatomical location of the transplanted kidney, pelvic bones might interfere with visualizing the stone and attenuating the shock waves generated by the machine. For these reasons, some authors propose to use the prone position during the treatments [12,20,47]. However, even when managing factors that decrease SWL efficacy, multiple treatments might be necessary since low-voltage, low-frequency SWL is recommended to have the lowest impact on the kidney parenchyma [24]. The graft might also be left with stone debris that might induce ureteral obstruction, occasionally silently, while passing through the ureter [48]. In our cohort, patients who were not stone-free after a single or multiple SWL underwent either PCNL (4/19) [21,22], fURS (1/19) [23]), or active surveillance for residual fragments <4–5 mm (14/19) [6,21]. Since most non-stone-free patients had residual fragments <5 mm and due to the variation in anatomy of the transplanted ureter, it is worth mentioning that tamsulosin was routinely given post-operatively only in one study [23] and that a ureteric stent was routinely placed only by another [19], suggesting therefore that adequate hydration and close monitoring of blood tests might be enough as post-operative care.

Since no clear indications regarding the treatment of urolithiasis in transplanted patients are present, we look at some evidence-based tips. The included studies suggest using SWL for stones <15 mm [5,19,20,23,24]. Stones should be treated as soon as possible, since some studies showed an increase in complication rates for patients waiting for these procedures [24]. During the procedure, patients should be placed in a prone position, and a water bag should be used to avoid compression and injury to the renal allograft, as it is usually superficial and pressed against the pelvic bone [19]. Low-voltage, low-frequency SWL (1–3 times) should be preferred, minimizing the effect on the renal allograft and reducing postoperative complications, such as hematuria and renal functional deterioration [19]. Arguments exist on whether to place a drain or not after the procedure since graft impairment is only transient and patients usually clear stones spontaneously [22,24]. However, all authors suggest following up patients closely for early detection of complications and performing a metabolic analysis of the stone for better surveillance monitoring and tailored management.

Our study has some limitations. First, all the included studies were retrospective in design and conducted on small samples. This bias is difficult to overcome since large prospective studies are difficult to perform because of the low incidence of allograft renal stones. Additionally, according to our search, the most recent papers were published in 2018, probably because, despite their clinical relevance, stones in allografts remain a relatively rare condition, and the reporting and academic publishing were likely to be affected due to the COVID pandemic. Second, we were not able to collect data on patients’ metabolic evaluation, nor were we able to stratify the stone analysis per procedure for each study. Lastly, due to the nature of the narrative studies, we lack some granular information, like the voltages and frequencies used to treat these patients, that might have differently influenced outcomes like SFR, complication rates, or secondary procedure rates. Nevertheless, the present study offers a good overview of the literature on a very specific subset of patients and some practical pitfalls that might be used in everyday clinics.

## 5. Future Directions

Since allograft urolithiasis is a rare condition and SWL is not routinely performed in all centers, centralization of care in specialized endourology centers where kidney transplantation is also carried out and these teams work collaboratively might improve treatment outcomes. Guidelines and algorithms can also provide management guidance [49,50]. In many cases, for larger stones, several sessions are needed to treat the stone. In this setting, better SWL machines with technological innovation aimed at optimizing stone treatment and reducing parenchymal injuries and the number of sessions could make this procedure more attractive. Finally, SWL in transplant is rare, and a central National or International registry would help collect data and improve outcomes. Emphasis should be placed on patient-reported outcome measures and the standardization of the stone-free rate definition [51].

## 6. Conclusions

SWL is an effective treatment option for transplanted patients with de-novo urolithiasis, offering good SFR without a high risk of major complications. However, management should consider patient and stone characteristics, the ability and expertise of the surgical team, and offer patient counseling and shared decision-making for choosing the most appropriate treatment.

## Figures and Tables

**Table 1:** Summary of included studies.

Article Details					Patient Characteristics		
Author	Year	Journal	Country	Level of Evidence	*n*	Male:Female	Age
Klinger	2002	*Urology*	Austria	4	5	NS	42.6 (32–71)
Li	2011	*Chinese Medical Journal*	China	4	7	5:2	34 (21–41)
Ferreira	2012	*Transplantation Proceedings*	Brazil	4	6	NS	45.6 (32–63)
Mahdavi	2014	*Experimental Clinical Trials*	Iran	4	10	7:3	37 (8–55)
Yuan	2015	*World Journal of Urology*	China	4	6	6:6	34.7 (11–58)
Brancherau	2017	*World Journal of Urology*	France	4	12	NS	48 (± 9)
Emiliani	2018	*European Urology*	Spain	4	22	NS	NS
Challacombe	2005	*BJU International*	UK	4	13	NS	42.3 (16–63)

NS: not specified.

**Table 2:** Outcomes and management of stones.

Author	Location	Size	Lithotripter	Stent or Nephrostomy	SFR	Complications	Management	Clavien
Klinger	1 ureteral 4 renal	7–15 mm	Piezolith 2500 (Wolf, Germany)	1	100.0%	Transient Hematuria (5/5)	Conservative	I
Li	5 ureteral 2 renal	NS	Xinyuan Company (Suzhou, China)	0	100.0%	0	-	-
Ferreira	NS	2–15 mm	NS	1	100.0%	0	-	-
Mahdavi	1 ureteral 9 renal	10–18 mm	NS	NS	70.0%	0	-	-
Yuan	3 ureteral 3 renal	12	NS	0	83.3%	UTI (3/6)	Antibiotics	II
Brancherau	NS	8–25 mm	NS	NS	50%	0	-	-
Emiliani	NS	NS	NS	0	52.9%	Transient hematuria (2/22) UTI (3/22) Steinstrasse (1/22)	Conservative Antibiotics NFS tube	I II IIIa
Challacombe	NS	<15 mm	Modulith (Storz, Germany)	9	100.0%	0	-	-

NS: not specified. UTI: urinary tract infection.

**Table 3:** Risk of bias analysis.

Certainty Assessment						Certainty
*n* of Studies	Study Design	Risk of Bias	Inconsistency	Indirecteness	Imprecision	
8	Retrospective observational studies	Very serious	Not serious	Very serious	Very serious	Very low

## Data Availability

All data are available in the studies included in the review and were discussed in the present manuscript.

## Supplementary Materials

The following supporting information can be downloaded at: https://www.mdpi.com/article/10.3390/jcm12134389/s1, Table S1: ISSM_MOOSE_Checklist.
